# Characterization of comorbidity heterogeneity among 13,667 patients with hidradenitis suppurativa

**DOI:** 10.1172/jci.insight.151872

**Published:** 2021-11-08

**Authors:** Vivian J. Hua, James M. Kilgour, Hyunje G. Cho, Shufeng Li, Kavita Y. Sarin

**Affiliations:** Department of Dermatology, Stanford University School of Medicine, Stanford, California, USA.

**Keywords:** Dermatology, Cardiovascular disease, Clinical practice, Skin

## Abstract

Hidradenitis suppurativa (HS) is a chronic, inflammatory skin disorder characterized by recurrent abscesses in the groin and flexural areas. HS is associated with a wide range of comorbidities that complicate the disease course. Although these comorbidities have been well described, it remains unclear how these comorbidities coassociate and whether comorbidity profiles affect disease trajectory. In addition, it is unknown how comorbidity associations are modulated by race and sex. In this comprehensive analysis of 77 million patients in a large US population–based cohort, we examined coassociation patterns among HS comorbidities and identified clinically relevant phenotypic subtypes within HS. We demonstrated that these subtypes not only differed among races, but also influenced clinical outcomes as measured by HS-related emergency department visits and cellulitis. Taken together, our findings provide key insights that elucidate the unique disease trajectories experienced by patients with HS and equip clinicians with a framework for risk stratification and improved targeted care in HS.

## Introduction

Hidradenitis suppurativa (HS), otherwise known as acne inversa, is a chronic, debilitating, and inflammatory skin disease affecting 0.7%–1.2% of the general US and European population (1). HS is characterized by inflammatory nodules or furuncles, deep abscesses, and draining sinus tracts that typically manifest in the intertriginous areas, resulting in purulent drainage and scarring (2–4). Hereditary cases of HS have been shown to be caused by mutations in the γ-secretase complex, affecting NOTCH signaling, but the exact pathogenesis of sporadic HS remains unclear (5, 6). Recent findings indicate that the primary defect in HS arises from follicular hyperkeratosis and occlusion, leading to follicular rupture and reactive inflammasome mediated by a complex cascade of cytokines, including IL-1 and TNF-α (1, 3, 6–8).

HS exhibits high disease heterogeneity, with a subset of patients experiencing only occasional inflammatory nodules and others developing extensive scarring and pain, leading to chronic infections, functional impairment, and decreased quality of life (1). Patients with HS also display marked heterogeneity in prognosis, response to treatment, and associated comorbidities. Race is one factor that has been associated with altered disease course, with previous research revealing that patients with color have significantly greater disease severity and health care utilization than White patients (9). Research has further demonstrated that individuals with HS are at increased risk of metabolic syndrome and autoimmune diseases as well as psychiatric conditions (10–22). Despite these known associations with HS, it remains unclear how these comorbidities associate with each other and whether these associations differ by race and sex (1).

This study investigates the spectrum of comorbidities associated with HS in a large US market claims data set encompassing over 77 million individuals. We investigated the impact of race and sex on the association of HS comorbidities and examined co-occurrence patterns among HS comorbidities.

## Results

### Cohort demographics.

The inclusion criteria yielded 13,667 patients with HS and 136,670 age-, sex-, race-, and enrollment-matched controls. Of the patients with HS, 10,054 (73.6%) were female and 3613 (26.4%) were male, with a mean age at first enrollment of 40.7 years and a standard deviation of 17.3 years. White patients comprised the majority of the HS cohort (60.1%); 20% of the patients with HS identified as Black, 11.4% identified as Hispanic, and 3.2% identified as Asian. The percentage of male patients within the Asian HS cohort (37.2%) was notably higher than the average percentage of male patients within the White, Black, and Hispanic HS cohorts (24.8%). The highest level of education attained by 11,338 (83%) patients with HS was less than a Bachelor’s degree compared with 107,373 (78.6%) of control patients. Last, 3316 (24.3%) of patients with HS utilized Medicare instead of commercial insurance (Supplemental Tables 3 and 4; supplemental material available online with this article; https://doi.org/10.1172/jci.insight.151872DS1).

### Comorbidities associated with HS, stratified by race and sex.

This analysis confirmed a number of previously identified comorbidities associated with HS. Individuals with HS were at significantly increased risk of all metabolic conditions included in the analysis, including hypertension, dyslipidemia, type 2 diabetes mellitus, obesity, myocardial infarction (MI), and cerebrovascular accident (CVA) (Table 1). Autoimmune conditions appeared in patients with HS at higher rates when compared with control subjects, including rheumatoid arthritis, ankylosing spondylitis, multiple sclerosis, inflammatory bowel disease, and psoriasis (Table 1). Female infertility was not significantly enriched in the HS cohort (OR 1.06, 95% CI 0.92–1.21), but polycystic ovary syndrome (PCOS) was (OR 2.03, 95% CI 1.85–2.22). Additionally, patients in the HS cohort were at increased risk of mental health disease, tobacco use, and substance use (ORs ranging from 2.05–3.65). Notably, cellulitis was enriched at a 6-fold rate in patients with HS compared with controls (OR 6.26, 95% CI 6.03–6.51), making cellulitis the second most increased comorbidity associated with HS, following pilonidal cyst (OR 13.71, 95% CI 12.11–15.52). Of the cutaneous malignancies, only the association with squamous cell carcinoma (SCC) was statistically significant (OR 1.27, 95% CI 1.10–1.46) (Table 1).

Notably, the enrichment of almost all comorbidities in HS was largely consistent across races and sex, with the exception of MI and CVA, for which logistic regressions unveiled a significant association of MI with HS in the White and Black HS cohort, but not the Asian and Hispanic HS cohort, as well as a significant association of CVA with HS in all racial cohorts with the exception of the Asian HS cohort. Additionally, as expected given the known carcinogenic role of UV radiation and the photoprotective effect of melanin, the OR of SCC in patients with HS when compared with non-HS controls was only significant in White patients (OR 1.38, 95% CI 1.18–1.61). Remarkably, the OR of SCC in patients with HS was significant in female patients (OR 1.44, 95% CI 1.20–1.72) but not male patients (OR 1.07, 95% CI 0.86–1.34) (Table 2).

### Pairwise correlations of comorbidities, clinical data, and severity in HS.

**Figure 1:**
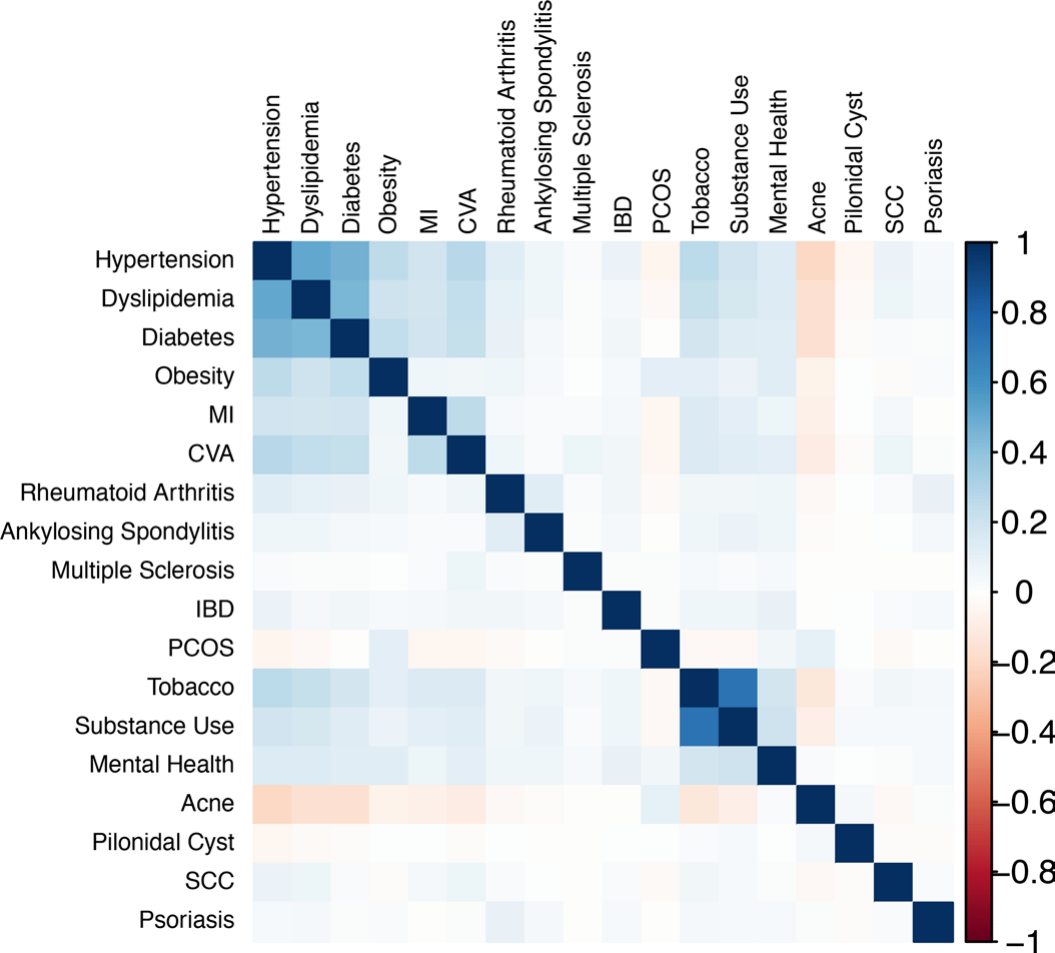
Pairwise association of comorbidities. An 18 × 18 matrix of the Pearson *r* correlation of each disease phenotype pair among 13,667 patients. Only correlations significant at *P* less than 0.01 were plotted. Strong pairwise correlations are displayed between members of metabolic syndrome, CVD, tobacco/substance use, and mental health.

To broadly understand how HS comorbidities associate with each other, pairwise Pearson correlations were performed between each pair of comorbidities (Figure 1). The 18 × 18 correlation matrix demonstrated tight pairwise correlations between all members of metabolic syndrome (hypertension, dyslipidemia, diabetes, obesity), MI, and CVA. This broad cluster of metabolic syndrome also exhibited strong pairwise correlations with tobacco, substance use, and mental health; together, all aforementioned comorbidities exhibited negative pairwise correlations with PCOS and acne.

### Clustering of comorbidities in HS.

**Figure 2:**
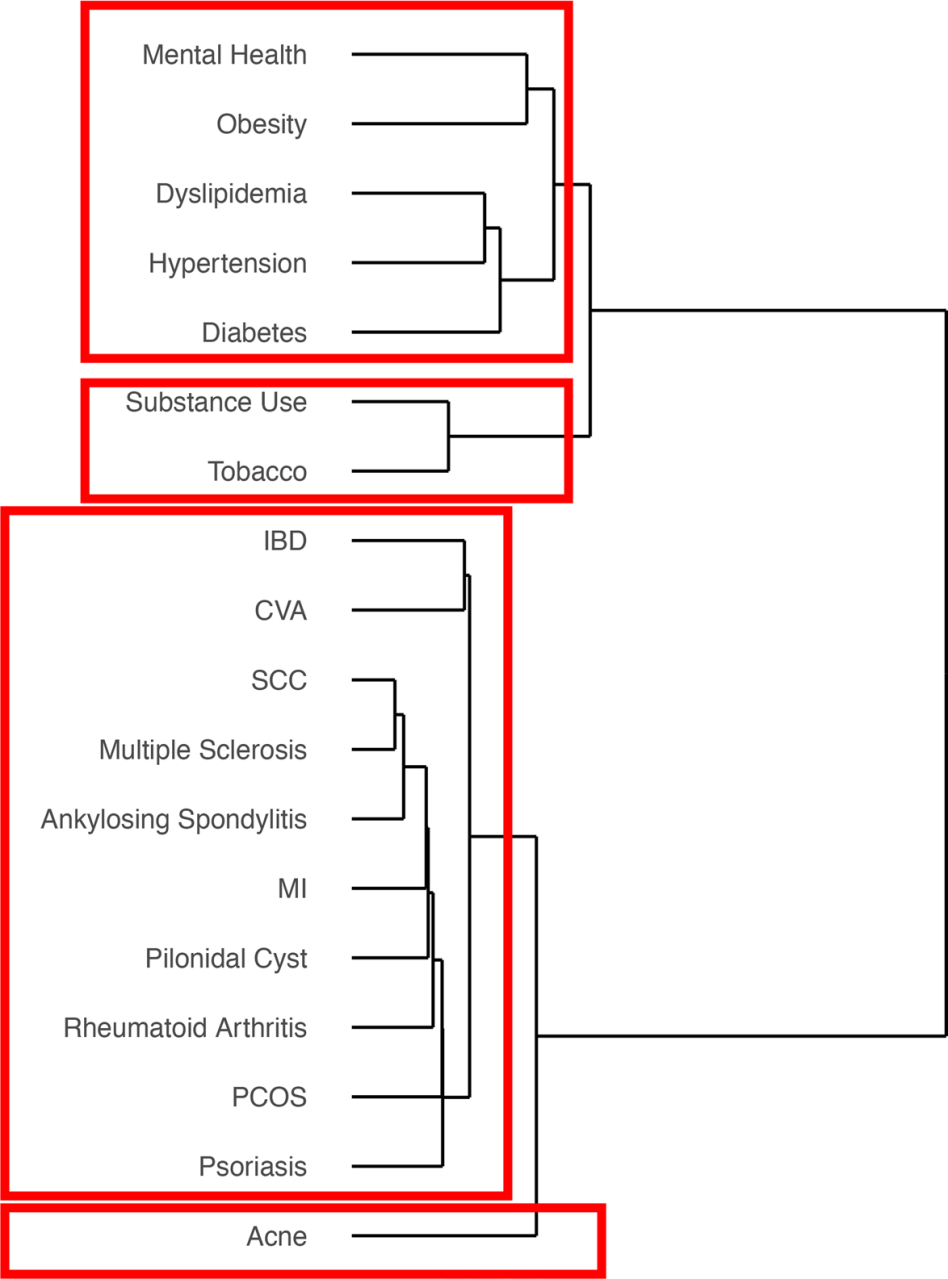
Clustering of comorbidities in HS. Unsupervised hierarchical clustering of comorbidities in HS was performed using Euclidean distance and Ward D linkage across 13,667 patients. Comorbidities were clustered according to similarity. Results again demonstrated tight clustering between members of metabolic syndrome, mental health, and tobacco/substance use.

Unsupervised hierarchical clustering of comorbidities in HS was performed using Euclidean distance and Ward D linkage. Consistent with the 18 × 18 Pearson correlation matrix, the results again demonstrated tight clustering between members of metabolic syndrome (dyslipidemia, hypertension, diabetes, obesity) and mental health. This cluster again formed a broader cluster with tobacco and substance use, which together composed one of the tightest independent clusters. Acne again formed a separate cluster (Figure 2).

### Clustering of patients in HS.

**Figure 3:**
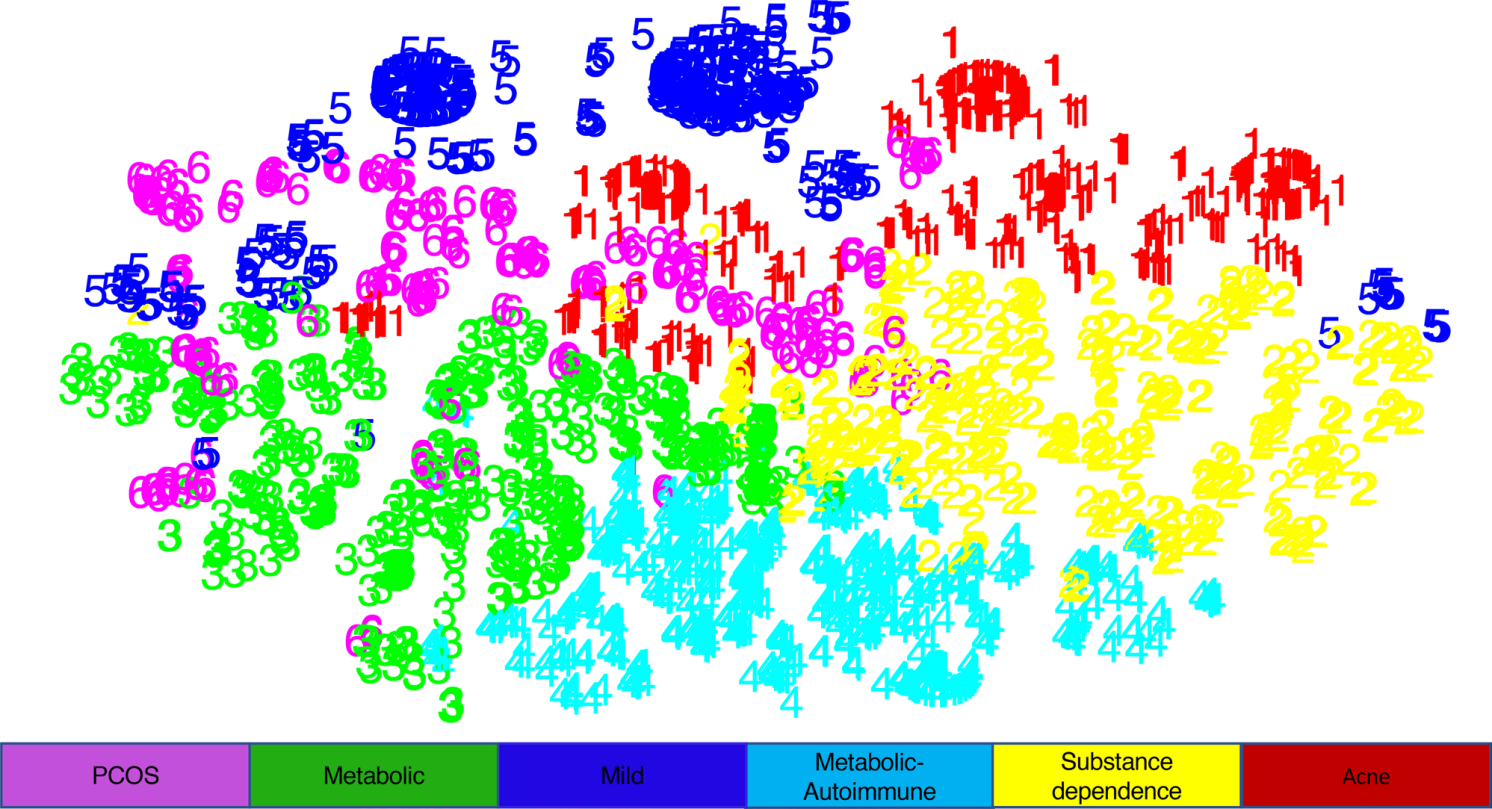
Patient clustering by comorbidity profile as visualized by t-SNE. K-means clustering was performed for 13,667 patients to create patient clusters, followed by dimensionality reduction and visualization with T-distributed stochastic neighbor embedding (t-SNE). This produced 6 clusters, which were named according to the defining comorbidities.

**Figure 4:**
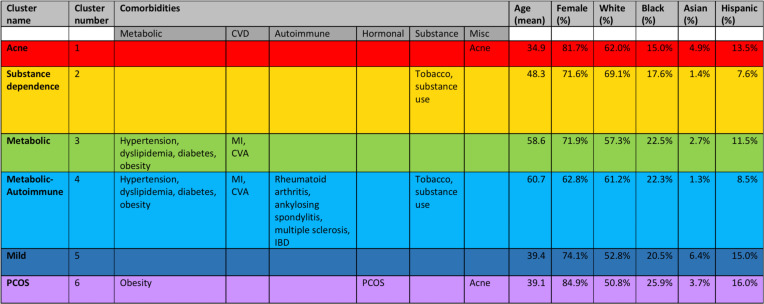
Patient clustering by comorbidity profile. K-means clustering was performed for 13,667 patients to create patient clusters, producing 6 clusters enriched in distinct comorbidity profiles and patient traits. Clusters were color-coded and subtypes were named according to the defining comorbidities. Percentages were calculated by dividing the patients belonging to each cluster with the comorbidity over the total number of patients in the cluster. A comorbidity is listed if that percentage was among the highest for that comorbidity across the 6 clusters.

In addition to the clustering of comorbidities in HS, the clustering of patients in HS was performed to identify patient subtypes within HS. Clustering was accomplished using K-means, followed by visualization of each cluster with T-distributed stochastic neighbor embedding (t-SNE). This produced 6 patient clusters enriched in distinct comorbidity profiles and patient demographics: a “Metabolic” cluster highly enriched in patients with metabolic syndrome; a “Metabolic-Autoimmune” cluster highly enriched in patients with metabolic syndrome, autoimmune comorbidities, and tobacco and substance use; a “Substance Dependence” cluster highly enriched only in patients with tobacco and substance use; an “Acne” cluster highly enriched in patients with acne; a “PCOS” cluster highly enriched in patients with PCOS, obesity, and acne; and a “Mild” cluster that was not highly enriched in patients with any of the comorbidities included in the analysis (Figures 3 and 4 and Supplemental Table 5).

The “Metabolic-Autoimmune” and “Metabolic” clusters consisted of the oldest patients (average age: 60.7 and 58.6 years, respectively). Additionally, the “Metabolic-Autoimmune” cluster was the most enriched in male patients (37.2%). The “Acne,” “PCOS,” and “Mild” clusters formed the youngest clusters (average age: 34.9, 39.1, and 39.4 years, respectively), while the “Acne” and “PCOS” clusters formed the most female clusters (81.7% and 84.9%, respectively).

The “PCOS” cluster was most enriched in Black patients (25.9%), followed by the “Metabolic-Autoimmune” and “Metabolic” clusters (22.3% and 22.5%, respectively). On the other hand, the “Mild” and “Acne” clusters were significantly more enriched in Asian patients (6.4% and 4.9%, respectively) compared with the other clusters (average 2.4%).

### Patient clusters stratified by HS severity and infectious complications.

Because measurements of HS severity and infectious complications are not recorded in claims data, they were proxied using HS-related emergency department (ED) visits and cellulitis, using a similar approach described in a prior study (23). Within the study period, 1591 (11.6%) patients with HS had an HS-related ED outpatient visit or admission. Multivariable logistic regression detected a 6-fold increased prevalence of cellulitis in patients with HS compared with controls (OR 6.26, 95% CI 6.03–6.51) (Table 1). The prevalence of cellulitis diagnoses was exceptionally high, occurring in 44.4% of the HS cohort. Cellulitis may therefore represent an infectious complication of HS or potentially a misdiagnosis of an HS flare.

Further examination uncovered that the prevalence of HS-related ED visits and cellulitis were enriched at different rates among the 6 phenotypic clusters. The “Metabolic-Autoimmune” cluster consisted of patients with the highest prevalence of HS-related ED visits (19.4%) and cellulitis diagnoses (61.7%). For both ED visits and cellulitis, this was subsequently followed by the “Metabolic” cluster (11.1% and 46.5%, respectively) and the “Substance Dependence” cluster (13.0% and 46.1%, respectively).

## Discussion

HS has a higher comorbidity burden than most inflammatory skin disorders, including psoriasis, affecting morbidity and mortality risk (24–26). Our regressions confirmed the association of 19 comorbidities with HS, including PCOS, metabolic syndrome, autoimmune disease, and mental health disorders (Table 1) (10–19, 21, 22, 27–33). In addition, our analysis identified a strong association between HS and psoriasis, which may be driven in part by the shared upregulation of TNF-α, IL-12/23, and IL-17 in both diseases (34). Furthermore, SCC is the main type of cutaneous malignancy reported in HS and additionally one of the most severe HS comorbidities, with a mortality rate of 50% in patients with HS (23). Our findings support enhanced clinician suspicion of atypical HS lesions or nonhealing wounds for this rare but frequently fatal comorbidity of HS.

The current understanding of race-specific prevalence in HS is limited by the overrepresentation of White cohorts in the majority of HS studies to date (1). However, HS is widely believed to disproportionately affect women and Black patients and has been associated with greater disease severity and health care utilization in the latter (1, 9, 35). Women represented the majority of our HS cohort (73.6%) but had relatively lower representation in our Asian HS cohort (62.8%). This relative decrease of affected women in Asian patients with HS is consistent with prior studies conducted in Korea and Taiwan, which revealed a male predominance in HS (20, 36–38) (Supplemental Table 3). Additionally, we confirmed a higher proportion of Black patients with HS (20%) in comparison with the proportion of Black patients within our database (10%) (Supplemental Table 3). Notably, however, conditional logistic regression demonstrated that the enrichment of almost all comorbidities in HS was largely consistent across races and sex, suggesting that race and sex are perhaps not as significant contributing factors to the association of HS with its comorbidities as has been previously postulated (Table 2 and Table 3). The exception in our findings was the association of MI with HS in the White and Black HS cohorts, but not the Asian and Hispanic HS cohorts, as well as the association of CVA with HS in all racial cohorts with the exception of the Asian HS cohort — likely a reflection of the fact that in the general population, age-adjusted coronary heart disease prevalence is lower among Asians (39). In addition, the association of HS with SCC was significant only in female patients with HS but not male patients, despite the incidence of SCC in males shown to be thrice the incidence of females in the general population — suggesting a critical role for the screening of atypical HS lesions in female patients with HS (40). Our findings therefore also confirm prior postulations that the pathophysiology of skin cancers in HS occurs in the setting of weakened cutaneous immunity and chronic inflammation, instead of the increased sun exposure for which the increased incidence of SCC in males in the general population has been attributed (23).

Patient clustering revealed 6 phenotypic subtypes in HS (Figure 3 and Figure 4). Notably, the “Metabolic-Autoimmune” cluster consisted of patients with the highest rates of HS-related ED visits and cellulitis diagnoses, suggesting more severe HS disease with increased complications. This was followed by patients in the “Metabolic” and “Substance Dependence” clusters. The reason for the association between these clusters and HS severity may be multifactorial. One possible explanation is a shared denominator in disease etiopathogenesis. In particular, the chronic systemic inflammation of HS pathophysiology, driven by elevated levels of IL-6 and TNF, not only contributes to HS severity but is also observed in all comorbidities enriched within these subtypes. Indeed, the association between HS and metabolic syndrome has been explained by the downstream secretion of inflammatory cytokines by adipose tissue, and circulating levels of proinflammatory mediators are known to drive atherosclerosis and thrombosis (27). Prior research also demonstrates that resting heart rate, which is linked to cardiovascular death, is significantly associated with severe HS (41). Furthermore, nicotine from tobacco smoking is known to modulate cutaneous immunity and increase epidermal hyperplasia and follicular occlusion, likely contributing to HS lesion formation and impaired healing (6). It is therefore possible that as levels of systemic inflammation rise, either due to or resulting in the associated comorbidities, HS severity rises as well. Additionally, we postulate that the association between the “Metabolic-Autoimmune” cluster and HS severity may also be attributed to the fact that this cluster is the oldest and most male cluster. Previous regression analyses have demonstrated that some of the most important characteristics associated with mortality and severity among patients with HS include age and male sex (25, 42).

To our knowledge, the only prior study that also characterized clinicopathological subsets of HS identified a 3-class model, in which phenotypes were characterized on the basis of lesion locations (axillary-mammary, follicular, and gluteal) instead of comorbidities (43). Notably, this study demonstrated that patients belonging to the follicular class were also more likely to be male and current smokers, with greater disease severity. These findings mirror our own findings of a phenotypic cluster within HS of predominantly male patients, enriched in tobacco use, who experience increased HS disease severity. While our dataset did not record the location of HS lesions, both our study and the prior study implicate the presence of a phenotypic cluster within HS separate from the typical axillary-mammary phenotype in a predominantly female population. Taken together, our study points to a potential role for the close clinical monitoring of older male patients with HS with a history of metabolic, autoimmune, and substance use comorbidities because these appear to be risk factors for severe HS. Further research is necessary to enhance our understanding of this at-risk group of patients with HS and to elucidate the direction of this association, as it is equally possible that severe HS itself is a risk factor for metabolic, autoimmune, and substance use comorbidities due to elevated levels of systemic inflammation.

We cannot discount the possibility that the patients in the younger clusters, including the “Acne,” “PCOS,” and “Mild” clusters, could develop metabolic syndrome or autoimmune disease as they age, but the distinct clinical demographics of each cluster nevertheless suggest that our study characterized unique subtypes within patients with HS. Of the 6 patient clusters we identified, the “PCOS,” “Metabolic-Autoimmune,” and “Metabolic” clusters were most enriched in Black patients with HS, a finding that likely reflects the increased prevalence of PCOS, diabetes, and cardiovascular disease in Black patients in the general population (Figure 4) (44–48). Additionally, the “Mild” and “Acne” clusters exhibited a nearly 3-fold and 2-fold increase, respectively, in the prevalence of Asian patients with HS when compared with the average of the other clusters (Figure 4). This suggests that a subset of Asian patients with HS demonstrate a milder comorbidity profile compared with patients with HS of other races, but it also suggests that a subset of Asian patients with HS is enriched in acne. Prior studies of HS in Asian-only cohorts have found particularly high associations of acne and pilonidal cyst with HS in Asian patients with HS (20, 36). Further research will need to be conducted to expand our understanding of the relationship between HS, acne, and pilonidal cyst in the Asian population. It is important for clinicians to be aware of this association in Asian patients because the risk of misclassification between these 3 diseases is high given their shared pathogenesis and clinical manifestations.

Our study benefited from the strong generalizability of a nationwide claims database across localities and races, as well as a large sample size, assuring sufficient power to detect statistically significant associations. Additionally, we utilized extensive clustering methodologies to fully characterize both comorbidity and patient clusters. However, our study is not immune to the limitations of cluster analysis. Cluster analysis can be applied to any data set, including random data, and assumes no underlying knowledge of the disease or measurement against a “correct solution.” Additionally, because claims data registries do not record information on HS severity, our assignment of HS severity was restricted to a binary system in which severe HS was determined using a previously published algorithm based on HS-related ED visits and inpatient admissions (23). The nature of database claims analysis also precluded our study from any determination of causality and made it vulnerable to potential errors in claims coding, such as overdiagnosis in order to generate higher claims and billings. Finally, US claims data sets may not fully capture the full spectrum of patients with HS and may bias toward those with less severe disease and sufficient health care access.

Our study comprehensively examined a large US population–based cohort of 77 million patients to investigate a spectrum of comorbidities associated with HS. In addition to confirming that patients with HS are at significantly increased risk of metabolic, autoimmune, and psychiatric comorbidities, this study characterized the variation in HS comorbidities by race and sex. Using potentially novel clustering methods, we not only investigated the coassociation patterns between HS comorbidities in the United States, but also demonstrated the clinical implications of HS phenotypic subtypes by examining clinical outcomes such as ED visits and cellulitis. These results provide clinicians with a framework with which to target risk stratification and early intervention for groups of patients within HS, thus facilitating a greater understanding of a debilitating skin disorder.

## Methods

### Data source.

Data were derived from a cross-sectional analysis using Clinformatics Data Mart database, one of the largest collections of deidentified patient-level data in the United States, containing individual-level health care claims information of more than 77 million patients with commercially insured and Medicare coverage from 2003 to June 2019. Available information encompasses patient socioeconomic status, member enrollment, and medical claims, including inpatient and outpatient services, facility claims, pharmacy claims, and lab results data.

### Cohort selection.

The HS cohort inclusion criteria consisted of a minimum of 3-year continuous enrollment time from January 2013 to June 2019, with a minimum of 2 International Classification of Diseases Clinical Modification (ICD-CM) codes: 9th revision (ICD-9-CM) code 705.83 or 10th revision (ICD-10-CM) code L73.2 for HS filed on different dates between January 2013 and June 2019. Prior studies have confirmed the validity of the ICD-9 diagnostic code for HS with chart validation and established a positive predictive value of 82% with 2 codes (49). A non-HS control group was also extracted in a 1:10 ratio matched for age, sex, and race. All controls were also matched to patients with HS by both start and end date of member coverage to ensure similar enrollment time. For each individual, a predefined set of comorbidities filed between January 2013 and June 2019 was extracted (Supplemental Table 1). Information on whether each patient with HS had a minimum of 1 code indicative of an HS-related ED visit within the study period was also extracted. For ED visits, HS must have been coded in the primary position or in the secondary position in conjunction with cellulitis, furuncles, or abscess in the primary position; additionally, the code must have occurred on the service date if outpatient or on the date of admission, date of discharge, or service date if inpatient. The codes used to define ED visits can be found in Supplemental Table 2.

### Statistics.

Conditional logistic regression was performed to estimate the OR of each comorbidity in patients with HS in comparison with age-, sex-, and race-matched non-HS controls in separate models. Conditional logistic regressions were also performed to estimate the ORs of each comorbidity in patients with HS in comparison with non-HS control patients for each race and sex in separate models. For the regressions for female infertility and PCOS, only females were included. Because obesity and tobacco use are known risk factors for hypertension, dyslipidemia, diabetes, MI, CVA, acne, and PCOS, the regressions for these comorbidities were also adjusted by obesity and tobacco use (50, 51).

To explore associations between comorbidities, pairwise Pearson correlations were calculated for each disease phenotype pair, and an 18 × 18 heatmap of *r* values was plotted to visualize associations significant at *P* values of less than 0.01.

To further understand how comorbidities cluster together, unsupervised hierarchical agglomerative clustering using Euclidean distance and Ward D linkage was performed to create the comorbidity clusters and dendrograms.

K-means clustering was performed to create patient clusters, followed by dimensionality reduction and visualization with t-SNE. K was chosen using an elbow plot. Clusters were color-coded and named according to defining comorbidities.

All data were analyzed using statistical software R (version 4.0.2, R Development Core Team, R Foundation for Statistical Computing) and SAS software (version 9.4, SAS Institute).

## Author contributions

VJH performed data acquisition, data analysis, manuscript writing, and manuscript editing. SL performed data acquisition, data analysis, and statistical supervision and assisted with manuscript revision. JMK performed data acquisition and assisted with manuscript revision. HGC performed data acquisition and assisted with manuscript revision. KYS designed and conceptualized the study, supervised data analysis, and revised the manuscript for intellectual content.

## Supplementary Material

Supplemental data

## Figures and Tables

**Table 3:**
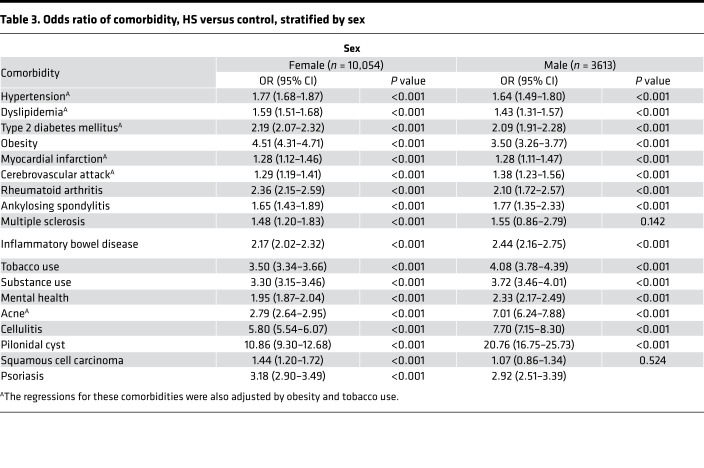
Odds ratio of comorbidity, HS versus control, stratified by sex

**Table 2:**
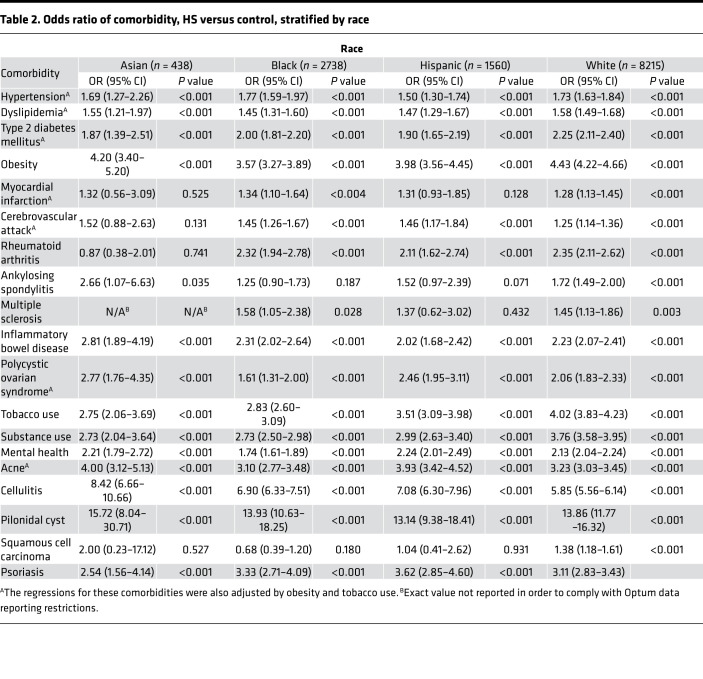
Odds ratio of comorbidity, HS versus control, stratified by race

**Table 1:**
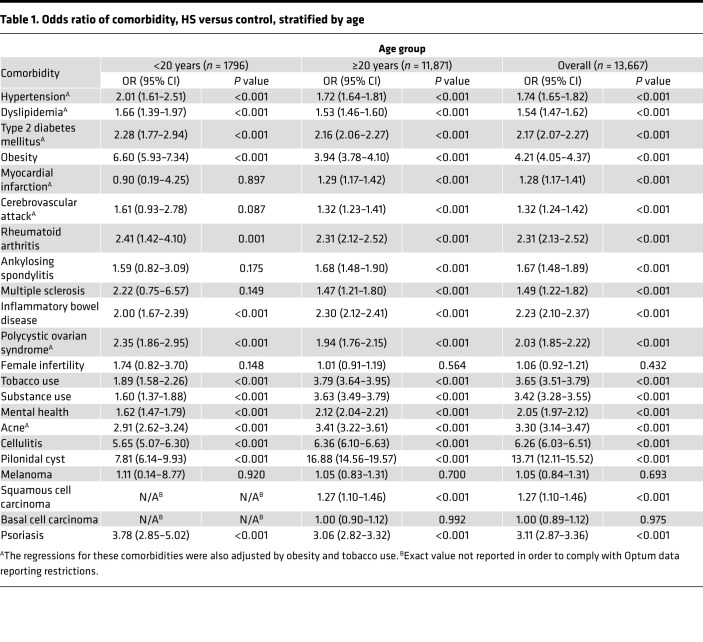
Odds ratio of comorbidity, HS versus control, stratified by age

## Version 1 09/21/2021

In-Press Preview

## Version 2 11/08/2021

Electronic publication
