# Supplementary material for: Characterization of comorbidity heterogeneity among 13,667 patients with hidradenitis suppurativa
Source: JCI Insight. 2021 Nov 8;6(21):e151872. doi: 10.1172/jci.insight.151872 (PMC8663549; doi:10.1172/jci.insight.151872)
Supplement: Supplemental data [file jciinsight-6-151872-s080.pdf]

Supplementary Table 1: ICD-9 and ICD-10 Codes

Comorbidity	ICD-10	ICD-9	Notes
Hypertension	I10-I16	401-405	
Dyslipidemia	E78	272	
Diabetes	E08-E13	249-250	
Obesity	E65-E68; Z68.3 - Z68.45; Z68.54	278.0-278.3; V85.3-V85.4; V85.54	
MI	I21-I24	410-412	
CVA	I60-I69	430-438	
Rheumatoid Arthritis	M06	714	
Ankylosing Spondylitis	M45	720	
Multiple Sclerosis	G35	340	
IBD	K50-K52	555-556	
PCOS	E28.2	256.4	
Female Infertility	N97	628.0 - 628.9	
Tobacco	Z72.0; Z87.891; F17.2	305.1; V15.82	
Substance Use	F10-F19; O99.32	303-305	*Exclude 305.1*
Mental Health	F01-F09; F20-F99	290-302; 306-319	*Excludes substance use disorders*
Acne	L70	706.0-706.1	
Cellulitis	L03	681-682	
Pilonidal Cyst	L05	685	
Cutaneous Melanoma	D03; C43	172.0-172.9	
SCC	C44.02; C44.12; C44.22; C44.32; C44.42; C44.52; C44.62; C44.72; C44.82; C44.92	173.02; 173.12; 173.22; 173.32; 173.42; 173.52; 173.62; 173.72; 173.82; 173.92	
BCC	C44.01; C44.11; C44.21; C44.31; C44.41; C44.51; C44.61; C44.71; C44.81; C44.91	173.01, 173.11, 173.21, 173.31, 173.41, 173.51, 173.61, 173.71, 173.81, 173.91	
Psoriasis	L40-L41	696.0-696.2; 696.8	

MI, myocardial infarction; CVA, cerebrovascular accident; IBD, inflammatory bowel disease; PCOS, polycystic ovary syndrome; SCC, squamous cell carcinoma; BCC, basal cell carcinoma

Supplementary Table 2: Codes Used to Define Emergency Department (ED) Visits

Outpatient and inpatient files	
CPT	99281-99285
Outpatient files	
PROCGRP	110, 111, 114
REVCODE	0450, 0451, 0452, 0456, 0459, 0981
STDPLAC	23
STDPROV	220, 428
SVCSCAT	10120, 10420, 10320, 10220, 12220, 20220, 10520, 20120, 21120, 21220, 22320, 22120, 30220, 30120, 30420, 30320, 30520, 30620, 31120, 31420, 31320, 31520, 31220, 31620
Inpatient files	
STDPLAC	23
STDPROV	220, 428
SVCSCAT	10120, 10420, 10320, 10220, 12220, 20220, 10520, 20120, 21120, 21220, 22320, 22120, 30220, 30120, 30420, 30320, 30520, 30620, 31120, 31420, 31320, 31520, 31220, 31620

Supplementary Table 3: Summary of HS Patient Demographics

	**Overall**	**Asian**	**Black**	**Hispanic**	**White**	**Unknown**
No. of Patients n (% of total cohort)	13667 (100%)	438 (3.2%)	2738 (20%)	1560 (11.4%)	8215 (60.1%)	716 (5.2%)
Gender *n (%)*						
Female	10054 (73.6%)	275 (62.8)	2141 (78.2)	1165 (74.7)	5967 (72.6)	506 (70.7)
Male	3613 (26.4%)	163 (37.2)	597 (21.8)	395 (25.3)	2248 (27.4)	210 (29.3)
Age at First Enrollment Mean (SD)	40.7 (17.3)	33.7 (14.1)	41.2 (16.8)	36.0 (16.7)	41.2 (17.3)	46.3 (18.7)
Total Years of Enrollment Mean (SD)	5.1 (1.2)	5.0 (1.2)	5.0 (1.3)	5.1 (1.2)	5.2 (1.2)	5.0 (1.3)
Education n (%)						
Less than 12th Grade	57 (0.4)	0 (0%)	<11[A]	>30[A]	15 (0.2)	<11[A]
High School Diploma	3931 (28.8)	>50[A]	1216 (44.4)	592 (38.0)	2002 (24.4)	>60[A]
Less than Bachelor's degree	7350 (53.8)	212 (48.4)	1365 (49.9)	767 (49.2)	4842 (58.9)	164 (22.9)
Bachelor's Degree Plus	1871 (13.7)	165 (37.7)	146 (5.3)	161 (10.3)	1322 (16.1)	77 (10.8)
Unknown	458 (3.4)	<11[A]	<11[A]	<11[A]	34 (0.4)	410 (57.3)
Household Income n (%)						
<$40K	3189 (23.3)	56 (12.8)	1102 (40.3)	378 (24.2)	1603 (19.5)	50 (7.0)
$40K-49K	951 (7.0)	26 (5.9)	239 (8.7)	137 (8.8)	524 (6.5)	25 (3.5)
$50K-59K	930 (6.8)	20 (4.6)	208 (7.6)	127 (8.1)	556 (6.8)	19 (2.7)
$60K-$74K	1219 (8.9)	27 (6.2)	241 (8.8)	172 (11.0)	763 (9.3)	16 (2.2)
$75K-$99K	1664 (12.3)	36 (8.2)	256 (9.4)	176 (11.3)	1156 (14.1)	40 (5.6)
$100K+	3194 (23.4)	168 (38.4)	253 (9.2)	280 (18.0)	2407 (29.3)	86 (12.0)
Unknown	2520 (18.4)	105 (24.0)	439 (16.0)	290 (18.6)	1206 (14.7)	480 (67.0)
Insurance Type n (%)						
Commercial	10351 (75.7)	402 (91.8)	1922 (70.2)	1300 (83.3)	6344 (77.2)	383 (53.5)
Medicare	3316 (24.3)	36 (8.2)	816 (29.8)	260 (16.7)	1871 (22.8)	333 (46.5)

[A]Exact value not reported in order to comply with Optum data reporting restrictions.

Supplementary Table 4: Summary of Control Patient Demographics

	Overall	Asian	Black	Hispanic	White	Unknown
No. of Patients n (% of total cohort)	136670 (100%)	4380 (3.2%)	27380 (20%)	15600 (11.4%)	82150 (60.1%)	7160 (5.2%)
Gender *n (%)*						
Female	100540 (73.6%)	2750 (62.8)	21410 (78.2)	11650 (74.7)	59670 (72.6)	5060 (70.7)
Male	36130 (26.4%)	1630 (37.2)	5970 (21.8)	3950 (25.3)	22480 (27.4)	2100 (29.3)
Age at First Enrollment Mean (SD)	40.7 (17.3)	33.7 (14.1)	41.2 (16.8)	36.0 (16.7)	41.2 (17.3)	46.3 (18.7)
Total Years of Enrollment Mean (SD)	5.2 (1.3)	5.2 (1.3)	5.2 (1.3)	5.2 (1.3)	5.2 (1.3)	5.2 (1.3)
Education n (%)						
Less than 12th Grade	627 (0.5)	18 (0.4)	77 (0.3)	404 (2.6)	108 (0.1)	20 (0.3)
High School Diploma	31150 (22.8)	471 (10.8)	9973 (36.4)	5445 (34.9)	14170 (17.3)	1091 (15.2)
Less than Bachelor's degree	75596 (55.3)	1994 (45.5)	14693 (53.7)	7785 (49.9)	47237 (57.5)	3887 (54.3)
Bachelor's Degree Plus	28881 (21.1)	1885 (43.0)	2557 (9.3)	1920 (12.3)	20385 (24.8)	2134 (29.8)
Unknown	416 (0.3)	12 (0.3)	80 (0.3)	46 (0.3)	250 (0.3)	28 (0.4)
Household Income n (%)						
<$40K	22089 (16.2)	399 (9.1)	8364 (30.6)	3019 (19.4)	9523 (11.6)	784 (11.0)
$40K-49K	7863 (5.8)	171 (3.9)	2319 (8.5)	1195 (7.7)	3849 (4.7)	329 (4.6)
$50K-59K	8164 (6.0)	202 (4.6)	2198 (8.0)	1310 (8.4)	4087 (5.0)	367 (5.1)
$60K-$74K	11781 (8.6)	346 (7.9)	2486 (9.1)	1547 (9.9)	6808 (8.3)	594 (8.3)
$75K-$99K	17952 (13.1)	517 (11.8)	2970 (10.9)	2071 (13.3)	11447 (13.9)	947 (13.2)
$100K+	47306 (34.6)	1866 (42.6)	4374 (16.0)	3562 (22.8)	34671 (42.2)	2833 (39.6)
Unknown	21515 (15.7)	879 (20.1)	4669 (17.1)	2896 (18.6)	11765 (14.3)	1306 (18.2)
Insurance Type n (%)						
Commercial	117405 (85.9)	4176 (95.3)	22709 (82.9)	14125 (90.5)	71090 (86.5)	5305 (74.1)
Medicare	19265 (14.1)	204 (4.7)	4671 (17.1)	1475 (9.5)	11060 (13.5)	1855 (25.9)

Supplementary Table 5: Complete Characteristics of HS Patient Clusters

**Cluster number**	**1**	**2**	**3**	**4**	**5**	**6**
**Cluster name**	**Acne**	**Substance dependence**	**Metabolic**	**Metabolic-Autoimmune**	**Mild**	**PCOS**
n	2527	2886	2828	2292	1947	1187
Age	34.9	48.3	58.6	60.7	39.4	39.1
Female	2065 (81.7%)	2065 (71.6%)	2034 (71.9%)	1439 (62.8%)	1443 (74.1%)	1008 (84.9%)
HS-related ED visits	166 (6.6%)	376 (13.0%)	314 (11.1%)	445 (19.4%)	187 (9.6%)	103 (8.7%)
Cellulitis	900 (35.6%)	1331 (46.1%)	1314 (46.5%)	1414 (61.7%)	652 (33.5%)	454 (38.3%)
White	1567 (62.0%)	1994 (69.1%)	1621 (57.3%)	1402 (61.2%)	1028 (52.8%)	603 (50.8%)
Black	380 (15.0%)	507 (17.6%)	635 (22.5%)	510 (22.3%)	399 (20.5%)	307 (25.9%)
Asian	123 (4.9%)	39 (1.4%)	77 (2.7%)	30 (1.3%)	125 (6.4%)	44 (3.7%)
Hispanic	341 (13.5%)	218 (7.6%)	324 (11.5%)	194 (8.5%)	293 (15.0%)	190 (16.0%)
Unknown	116 (4.6%)	128 (4.4%)	171 (6.0%)	156 (6.8%)	102 (5.2%)	43 (3.6%)
Hypertension	117 (4.6%)	1264 (43.8%)	2521 (89.1%)	2156 (94.1%)	123 (6.3%)	647 (54.5%)
Dyslipidemia	343 (13.6%)	1260 (43.7%)	2742 (97.0%)	2129 (92.9%)	319 (16.4%)	181 (15.2%)
Diabetes	90 (3.6%)	107 (3.7%)	1579 (55.8%)	2162 (94.3%)	91 (4.7%)	139 (11.7%)
Obesity	757 (30.0%)	1647 (57.1%)	2091 (73.9%)	1901 (82.9%)	710 (36.5%)	1157 (97.5%)
MI	7 (0.3%)	54 (1.9%)	137 (4.8%)	420 (18.3%)	5 (0.3%)	8 (0.7%)
CVA	46 (1.8%)	151 (5.2%)	412 (14.6%)	708 (30.9%)	26 (1.3%)	30 (2.5%)
Psoriasis	127 (5.0%)	210 (7.3%)	192 (6.8%)	178 (7.8%)	85 (4.4%)	72 (6.1%)
Rheumatoid Arthritis	55 (2.2%)	148 (5.1%)	212 (7.5%)	226 (9.9%)	37 (1.9%)	43 (3.6%)
Ankylosing Spondylitis	24 (0.9%)	85 (2.9%)	59 (2.1%)	107 (4.7%)	15 (0.8%)	11 (0.9%)
Multiple Sclerosis	13 (0.5%)	35 (1.2%)	24 (0.8%)	29 (1.3%)	5 (0.3%)	9 (0.8%)
IBD	210 (8.3%)	340 (11.8%)	331 (11.7%)	354 (15.4%)	99 (5.1%)	106 (8.9%)
PCOS	74 (2.9%)	136 (4.7%)	123 (4.3%)	97 (4.2%)	29 (1.5%)	351 (29.6%)
Tobacco	98 (3.9%)	2804 (97.2%)	412 (14.6%)	2169 (94.6%)	146 (7.5%)	105 (8.8%)
Substance use	94 (3.7%)	2738 (94.9%)	66 (2.3%)	1876 (81.8%)	39 (2.0%)	30 (2.5%)

Mental health	1999 (79.1%)	1970 (68.3%)	1643 (58.1%)	1870 (81.6%)	0 (0.0%)	409 (34.5%)
Acne	1378 (54.5%)	604 (20.9%)	325 (11.5%)	259 (11.3%)	0 (0.0%)	644 (54.3%)
Pilonidal Cyst	149 (5.9%)	162 (5.6%)	72 (2.5%)	104 (4.5%)	73 (3.7%)	44 (3.7%)
SCC	11 (0.4%)	57 (2.0%)	83 (2.9%)	64 (2.8%)	14 (0.7%)	9 (0.8%)

MI, myocardial infarction; CVA, cerebrovascular accident; IBD, inflammatory bowel disease; PCOS, polycystic ovary syndrome; SCC, squamous cell carcinoma

Supplementary Table 6: Percentage of Patients with Metabolic or Autoimmune Conditions[A]

Cluster name	Cluster number	Metabolic	Autoimmune
Acne	1	7.1%	11.2%
Substance dependence	2	52.2%	18.7%
Metabolic	3	100.0%	19.7%
Metabolic-Autoimmune	4	100.0%	27.0%
Mild	5	11.6%	7.8%
PCOS	6	66.0%	13.4%

[A]A patient was considered to have a “metabolic” condition if the patient had at least 2 of the following: hypertension, dyslipidemia, diabetes, obesity, myocardial infarction (MI), or cerebrovascular accident (CVA) (chosen because metabolic syndrome is often defined as 2 or more of its associated comorbidities). A patient was considered to have an “autoimmune” condition if the patient had at least 1 of the following: rheumatoid arthritis, ankylosing spondylitis, multiple sclerosis, or inflammatory bowel disease (IBD).
